# Biologically active withanolides from *Physalis peruviana*

**DOI:** 10.1080/13880209.2025.2488136

**Published:** 2025-04-26

**Authors:** Mayuramas Sang-Ngern, Ashley Fukuchi, Tamara P. Kondratyuk, Eun-Jung Park, Charles J. Simmons, Marisa M. Wall, Sam E. Lorch, John M. Pezzuto, Leng Chee Chang

**Affiliations:** ^a^ Department of Pharmaceutical Sciences, The Daniel K. Inouye College of Pharmacy, University of Hawai’i at Hilo, Hilo, HI, United States; ^b^ School of Cosmetic Science, Mae Fah Luang University, Tasud, Muang District, Chiang Rai, Thailand; ^c^ College of Pharmacy and Health Sciences, Western New England University, Springfield, MA, United States; ^d^ X-ray Diffraction Laboratory, Department of Chemistry, University of Hawai’i at Hilo, Hilo, HI, United States; ^e^ Daniel K. Inouye U.S. Pacific Basin Agricultural Research Center, Hilo, HI, United States; ^f^ Lani ko Honua Berry Farm, Hilo, HI, United States

**Keywords:** Novel and chlorinated withanolides, *Physalis peruviana*, anti-inflammatory, cape gooseberry, poha, goldenberry

## Abstract

**Context:**

*Physalis peruviana* L. (Solanaceae), also known as Poha, has been used in traditional medicine since pre-Columbian times, particularly in treating cancer.

**Objective:**

To study the chemical composition and potential medicinal properties of Poha.

**Materials and Methods:**

The fresh fruits and aerial parts of Poha were extracted. The isolation of extract yields a novel withanolide (physaperuvin K; **1**) from the edible fruit, and seven withanolides (**2**–**8**), including a rare chlorinated withanolide (physalolactone; **2**) from the aerial parts. Structure elucidation/determination was performed, some acetate derivatives were prepared (**2a**–**6a**), and the compounds were evaluated with *in vitro* assays indicative of anti-inflammatory activity.

**Results:**

The structure of **1** was elucidated through NMR spectroscopic analyses. The absolute configuration of compound **2** was determined using single-crystal X-ray diffraction. Compounds **1**, **2**, and **3** exhibited inhibition of tumor necrosis factor-α-induced nuclear factor-kappa B (NF-κB) activity with IC_50_ values of 10, 60, and 40 nM, respectively, without causing cytotoxicity at a concentration of 50 μM. Furthermore, compounds **1**–**3** reduced nitric oxide (NO) production in lipopolysaccharide-activated RAW 264.7 mouse macrophage cells with IC_50_ values ranging from 0.32 to 13.3 μM without overt cytotoxicity. Overall, acetylation did not significantly impact activity, except for compound **4**, wherein the IC_50_ values in the NF-κB and NO assays were reduced from 11.0 to 0.33 μM, and 1.8 to 0.24 μM, respectively.

**Conclusions:**

These findings enhance our understanding of Poha’s constituents and potential medicinal properties. One of the most bioactive compounds identified in this study, physaperuvin K, is found in edible fruit.

## Introduction

Withanolides are a group of highly oxygenated C_28_ ergostane-type phytosterols that are found in Solanaceae plants in the genera *Acnistus* Schott (1829)*, Datura* L. (1753), *Dunalia* Kunth (1818), *Jaborosa* Juss. (1789)*, Lycium* (1753)*, Physalis* L. (1753)*, Tubocapsicum* (Wettst.) Makino (1908), and *Withania* Pauquy (1825) (Glotter 1991; Pigatto et al. 2014). They are classified into four major groups: 20-deoxy withanolides, 20-hydroxy withanolides, withanolides with the C-17-α side chain-orientation, and withanolides in which the basic skeleton is modified or chlorinated. In recent years, withanolides have attracted significant scientific interest due to their various biological activities, including anti-cancer (Sakarkar and Deshmukh 2011; Merina et al. 2012), anti-microbial (Budhiraja et al. 2000; Abou-Douh 2002; Kharel et al. 2012), and anti-inflammatory effects (Wang et al. 2012). Such studies have shown that withanolides have promising potential as lead compounds with novel anti-inflammatory properties.

*Physalis peruviana*, also known as Poha, belongs to the Solanaceae family (Pigatto et al. 2014; Medina et al. 2015). *P. peruviana* has been used in Ayurvedic medicine and several countries, including Cameroon, Colombia, India, Kenya, Nepal, and South Africa. Different parts of the plant such as the leaves, fruits, whole plants, roots, stems, and seeds are used in traditional medicine. It is used to treat ailments including asthma, cancer, dermatitis, gastrointestinal disorders, hepatitis, and fungal and bacterial infections (Kasali et al. 2021). It has been used in traditional medicine since pre-Columbian times (Medina et al. 2015), particularly in the treatment of cancer (Lan et al. 2009; Medina et al. 2015; Tammu and Ramana 2015). This herbaceous, perennial plant has ribbed, purplish branches and velvety, heart-shaped, serrated leaves. The fruits are globose berries with smooth, waxy, orange-yellow skin and sweet, slightly acidic pulp. The fruits are encased in straw-colored husks (Morton 1987). They are rich in vitamin C and have antioxidant activity (Ramadan and Moersel 2007; Sathyadevi and Subramanian 2015). The fruits are consumed fresh or processed as jams. Previous phytochemical studies have shown that the fruits contain polyphenols such as quercetin, myricetin, and kaempferol (Licodiedoff et al. 2013; Sathyadevi and Subramanian 2015). *In vitro* studies with withanolides from *P. peruviana* (aerial part) showed anti-proliferative effects with human lung cancer cells (H1299) (Yen et al. 2010), anti-hepatotoxic effects with HepG2 cells (Wu et al. 2004) and cytotoxicity against lung [A549 (Lan et al. 2009) and NCl-H460 (Choudhary et al. 2010)] breast (MDA-MB-231 and MCF7), and liver (HepG2 and Hep3B) cancer cell lines (Lan et al. 2009).

Withaferin A, for example, displays anti-cancer and anti-inflammatory activities through mechanisms that modulate NF-κB (Ichikawa et al. 2006; Wang et al. 2012). A previous study demonstrated withanolides act as Michael addition acceptors, conjugate with glutathione, and lead to the inhibition of NF-κB (Ding et al. 2014). Pro-inflammatory mediators, such as nitric oxide (NO) and prostaglandins (PGs) in tumor cells and tissues, enable carcinogenesis progression (Choudhari et al. 2013). Moreover, NO production by inducible nitric oxide synthase (iNOS) is associated with chronic inflammation, a hallmark of cancer development and progression. The up-regulated production of NO is associated with early tumor development (Park and Pezzuto 2002). Taken together, the inhibition of TNF-α-activated NF-κB and NO production in lipopolysaccharide (LPS) activated murine macrophages RAW 264.7 cells were used as models for examining withanolides as possible chemotherapeutic/anti-inflammatory agents.

Despite the more extensive information regarding anti-cancer activity, there are only a few reports regarding the anti-inflammatory activities of naturally occurring withanolides from *P. peruviana* (Chang et al. 2016; Park et al. 2016; Sang-Ngern et al. 2016; Xu et al. 2017; Park et al. 2019). In our ongoing search for bioactive compounds derived from nature, *P. peruviana* was a lead for further investigation. As reported herein, a series of withanolides were isolated from *P. peruviana*. In addition, some of the withanolide isolates were used to prepare acetylated derivatives to explore their biological activity. Acetylation contributes to the bioactivity of many important natural medicinal products: converting the polar hydroxyl group into an acetyl group can enhance bioavailability. The resulting substances were tested in cancer chemopreventive/anti-inflammatory bioassays (Park et al. 2016, 2019) with promising results.

## Materials and methods

### General experimental procedures

Fourier Transform Infrared (FT-IR) spectra were recorded on a Bruker Tensor-27 spectrometer, while UV spectra were recorded using a Shimadzu PharmaSpec-1700 UV-visible spectrophotometer (Shimadzu Corporation, Kyoto, Japan). Optical rotations were measured on a Rudolph Research Autopol IV multiwavelength polarimeter (Rudolph Research Analytical, Hackettstown, NJ, USA**).** Electronic circular dichroism (ECD) spectra were obtained with a JASCO J-815 spectropolarimeter (JASCO Inc., Tokyo, Japan). Nuclear Magnetic Resonance (NMR) spectra were acquired on a Bruker AVANCE DRX-400 MHz spectrometer, which was equipped with a 5 mm BBFO Z-gradient smart probe featuring Automatic Tune and Match (Bruker, Billerica, MA, USA). Residual solvent was used as the standard, and the spectra were processed using MestReNova 14.2 software. High-resolution accurate mass data were collected with an Agilent 6530 Accurate-Mass Q-TOF LC-MS (electrospray ionization) mass spectrometer (Santa Clara, CA, USA). HPLC grade solvents were purchased from VWR (VWR Chemicals, Atlanta, GA, USA) or were of LC-MS grade (Optima LC/MS, Fisher Scientific, Zaventem, Belgium). Thin-layer chromatography (TLC) was performed on precoated 60 F_254_ 0.25 mm glass-backed plates (Sorbent Technologies, Atlanta, GA, USA), visualization was achieved using UV light at 254 nm or by staining with a 10% sulfuric acid-vanillin solution Column chromatography was conducted on silica gel (230–400 mesh, Sorbent Technologies, Atlanta, GA, USA), and gel filtration was carried out using Sephadex LH-20 (GE Healthcare, Piscataway, NJ, USA). Semi-preparative HPLC was performed on a Shimadzu-LC20 HPLC system equipped with a photodiode array detector, utilizing a YMC reversed-phase C_18_ chiral column (150 × 20 mm, 5 *μ*m), with a flow rate of 3.0 mL/min.

### Plant material

In September 2011, the aerial parts and fresh fruits of *P. peruviana* were collected from Lani ko Honua Berry Farm in Pepeekeo, Hawaii, USA. The collected plant materials parts were separated and thoroughly cleaned to remove dust and soil before extraction. The plant was identified by Dr. Marisa Wall, and a voucher specimen (No. PPJ002) was deposited at the Natural Product Chemistry Laboratory at the Daniel K. Inouye College of Pharmacy, University of Hawai’i at Hilo, Hilo, HI, United States.

### Extraction and isolation

Approximately 10 kg of fresh fruits from *P. peruviana* were extracted using 70% aqueous ethanol (8 L) three times at room temperature, with each extraction occurring overnight. The extract was then filtered, and the supernatant was collected and concentrated by evaporating the solvent using a rotary evaporator. This process resulted in a crude extract weighing 406.5 g, which was then suspended in 500 mL of distilled water. Next, the extract was subjected to successive partitioning with *n*-hexane, ethyl acetate, and *n*-butanol in that order. The *n*-BuOH-soluble fraction, weighing 156.24 g, was further analyzed using a gel filtration chromatographic column (CC) (Sephadex LH-20) (CC) and eluted with a gradient from 100% water to 0% methanol, resulting in six major fractions labeled PFB 1–6. Fraction PFB3, weighing 2.38 g, was then further fractionated on silica gel CC (200–300 mesh) and eluted with a CHCl_3_−MeOH mixture (ranging from 100:0 to 60:40) to isolate compound **2**. Fraction PFB6 was purified using medium-pressure liquid chromatography (MPLC) over an RP C_18_ column and eluted with a mixture of H_2_O and MeOH (from 100:0 to 0:100) to obtain compound **1** (18 mg).

Approximately 27 kg of aerial parts of *P.peruviana* were collected, dried, ground, and extracted with methanol (100 L) three times at room temperature. Each extraction was conducted overnight. After filtering, the supernatant was concentrated using rotary evaporation under reduced pressure, resulting in a brownish crude extract weighing 1.29 kg. The soluble extract was then partitioned sequentially with *n*-hexane, ethyl acetate, and *n*-butanol. The ethyl acetate-soluble fraction (165.9 g) was subjected to column chromatography (CC) over a silica gel (4 kg, 450–550 mesh). The chromatography used a CHCl_3_ solvent system with increasing amounts of methanol, ranging from 100:0 to 60:40, yielding 13 fractions (FA_1–13_). Subfraction FA_4_ (63.5 g) underwent CC on Si gel (2.5 kg, 450–550 mesh), eluting with CHCl_3_−MeOH gradient from 100:0 to 60:40, yielding 12 distinct fractions (FA_4.1 − 4.12_). Fraction FA_4.12_ (52.8 g) was purified through repeated silica gel chromatography using the same gradient, resulting in five subfractions (FA_4.12.1–12.5_). From fraction FA_4.12.3_, compound **6** (15 mg) was isolated. Subfraction FA_6_ was similarly separately using silica gel CC under the same CHCl_3_−MeOH gradient, leading to the isolation of compound **5** (90 mg). Subfraction FA_10_ (24.4 g) was subjected to column chromatography on silica gel CC (40–75 mesh), utilizing the CHCl_3_−MeOH (from 100:0 to 60:40) gradient, which produced ten fractions (FA_10.1–10.10_). Subfraction FA_10.5_ (50 mg) was further purified using semi-preparative high-performance liquid chromatograph (HPLC) using a YMC PackPro C_18_ column (150 × 20 mm, 5 *µ*m) eluting with MeCN − H_2_O (from 30:70 to 100:0) at a flow rate of 1 mL/min, resulting in the successful isolation of compounds **7** (6.0 mg, *t*_R_ 54.08 min) and **8** (4.5 mg, *t*_R_ 55.17 min). Subfraction FA_12_ (5 g) was subjected to gel filtration Sephadex LH-20 CC and eluted with H_2_O − MeOH from 100:0 to 0:100, yielding fractions FA_12.1–12.10_. Subfraction FA_12.8_ (245.0 mg) was finally purified *via* semi-preparative HPLC (reversed phase C_18_ column, 150 × 20 mm, 5 *µ*m) eluting with MeOH − H_2_O (from 50:50 to 100:0) at a flow rate 2 mL/min, to obtain compounds **3** (25.12 mg) and **4** (112.5 mg).

Physaperuvin K (**1**): yellowish solid; [α]^25^_D_ +390° (*c* 0.1, MeOH); CD (*c* 0.05, MeOH) 250 (+865.25); UV (MeOH) λ_max_ (log ɛ) 206 (0.80) nm; IR ν_max_ 3335, 1682 cm^−1^; ^1^H and ^13^C NMR data, see Table 1; HRESIMS *m/z* 1115.5175 [2M + K]^+^ (calcd for C_56_H_84_O_20_K, 1115.5193) (Figure S16, Supporting Information).^1^H NMR (CD_3_OD, 400 MHz) *δ*_H_ 4.81 (1H, dd, *J* = 13.2, 3.9 Hz, H-22), 4.29 (1H, d, *J* = 6.4 Hz, H-4), 4.23 (1H, m, H-3), 4.08 (1H, dd, *J* = 6.9, 2.1 Hz, H-6), 2.57 (1H, m), 2.65 (1H, m), 2.51 (1H, m), 2.39 (1H, dd, *J* = 18.0, 3.5 Hz, H-2), 1.96 (3H, s, H-28), 1.84 (3H, s, H-27), 1.37 (3H, s, H-21), 1.24 (3H, s, H-19), 1.09 (3H, s, H-18).^13^C NMR (CD_3_OD, 100 MHz) *δ*_C_ 213.4 (C-1), 169.3 (C-26), 153.5 (C-24), 122.1 (C-25), 88.8 (C-17), 83.8 (C-14), 83.2 (C-22), 80.0 (C-20), 78.8 (C-6), 78.3 (C-4), 77.5 (C-5), 74.8 (C-3), 56.1 (C-10), 55.9 (C-13), 42.8(C-2), 37.7 (C-16), 35.8 (C-8), 35.3 (C-9), 34.6 (C-23), 33.2 (C-15), 31.6 (C-12), 27.1 (C-7), 22.1 (C-11), 21.0 (C-28), 20.7 (C-18), 19.6 (C-21), 15.5 (C-19), 12.5 (C-27). The 1D and 2D NMR spectra of physaperuvin K (**1**) are presented in Figure S2–S7, Supporting Information.

**Table 1.** ^1^H And ^13^C NMR spectroscopic data of compounds **1** and **2**.

No.	(**1**)*^a^*		(**2**)*^a^*	
	*δ*_H, mult._ (*J* in Hz)	*δ*_C_	*δ*_H, milt._ (*J* in Hz)	*δ*_C_
1		213.4		203.6
2	2.39 dd (18.0, 3.5), 2.57 m	42.8	5.89 dd (10.3, 2.1)	126.9
3	4.23 m	74.8	6.53 dd (10.3, 2.6)	147.3
4	4.29 d (6.4)	78.3	4.99 t (2.4)	65.7
5		77.5		79.8
6	4.08 dd (6.9, 2.1)	78.8	4.35 dd (9.6, 7.9)	65.8
7	1.79, 2.03 m	27.1	1.55, 2.53 m	37.2
8	2.09 m	35.8	2.13	35.5
9	2.10 m	35.3	2.16	39.9
10		56.1		58.1
11	1.33, 1.59 m	22.1	1.33, 0.86 m	23.6
12	1.28, 2.19 m	31.6	2.16, 1.21 m	31.0
13		55.9		55.6
14		83.8		83.2
15	1.53, 1.69 m	33.2	1.50, 1.74 m	32.9
16	1.55, 2.55 m	37.7	2.04, 2.13 m	39.6
17		88.8		88.0
18	1.09 s	20.7	1.04	21.2
19	1.24 s	15.5	1.21	9.9
20		80.0		79.5
21	1.37 s	19.6	1.36 s	19.3
22	4.81 dd (13.2, 3.9)	83.2	4.76 dd (13.2, 3.8)	82.2
23	2.51, 2.65 m	34.6	2.51, 2.61 m	35.3
24		153.5		153.2
25		122.1		121.8
26		169.3		168.9
27	1.84 s	12.5	1.81 s	12.5
28	1.96 s	21.0	1.92 s	20.9

Spectra recorded at ^13^C NMR (100 MHz) in ^a^CD_3_OD, Chemical shift (*δ*) are in ppm, and coupling constants (*J* in Hz) are given in parentheses. The assignments were based on DEPT, COSY, NOESY, HSQC, and HMBC experiments.

Physalolactone (**2**): colorless needles; [α]^25^_D_ +216^ο^ (*c* 0.1, MeOH); CD (*c* 0.05, MeOH) 236 (+992.81); UV (MeOH) λ_max_ (log ɛ) 213 (0.48) nm; IR ν_max_ 3311, 1675 cm^−1^; ^1^H NMR data and ^13^C NMR data, see Table 1; HRESIMS *m/z* 561.2224 [M + Na]^+^ (calcd for C_28_H_39_ClNaO_8_, 561.2231) (Figure S17, Supporting Information). ^1^H NMR (CD_3_OD, 400 MHz) *δ*_H_ 6.53 (1H, dd, *J* = 10.3, 2.6 Hz, H-3), 5.89 (1H, dd, *J* = 10.3, 2.1 Hz, H-2), 4.99 (1H, t, *J* = 2.4 Hz, H-4), 4.76 (1H, dd, *J* = 13.2, 3.8 Hz, H-22), 4.35 (1H, dd, *J* = 9.6, 7.9 Hz, H-6), 1.92 (3H, s, H-28), 1.81 (3H, s, H-27), 1.36 (3H, s, H-21), 1.21 (3H, s, H-19), 1.04 (3H, s, H-18). ^13^C NMR (CD_3_OD, 100 MHz) *δ*_C_ 203.6 (C-1), 168.9 (C-26), 153.2 (C-24), 147.3 (C-3), 126.9 (C-2), 121.8 (C-25), 88.0 (C-17), 83.2 (C-14), 82.2 (C-22), 79.8 (C-5), 79.5 (C-20), 65.8 (C-6), 65.7 (C-4), 58.1 (C-10), 55.6 (C-13), 39.9 (C-9), 39.6 (C-16), 37.2 (C-7), 35.5 (C-8), 35.3 (C-23), 32.9 (C-15), 31.0 (C-12), 23.6 (C-11), 21.2 (C-18), 20.9 (C-28), 19.3 (C-21), 12.5 (C-27), 9.9 (C-19). The 1D and 2D NMR spectra of physalactone (**2**) are shown in Figure S8–15 of the Supporting Information).

#### Single-crystal X-ray structure analysis of physalolactone

**Figure 1.**
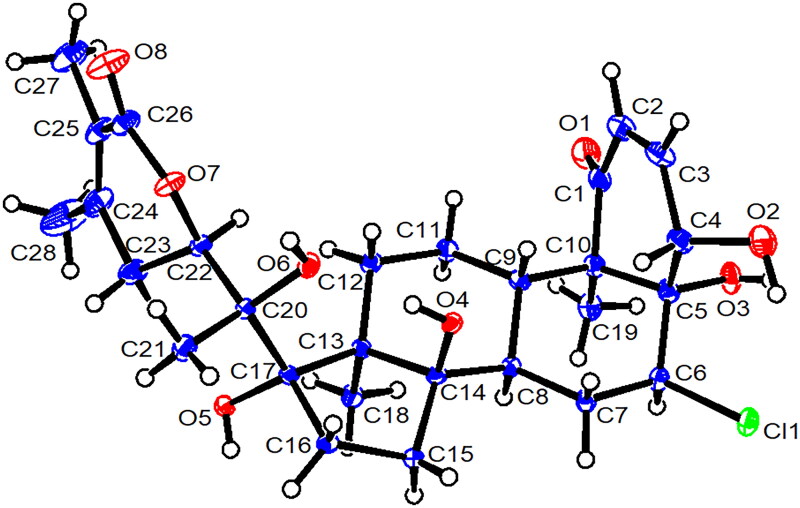
ORTEP drawing of physalolactone (**2**); C_28_H_39_ClO_8_⋅H_2_O. Displacement ellipsoids are drawn at the 50% probability level at 120 K; the crystalline H_2_O molecule is not shown. Absolute configuration is: C4(*S*), C5(*R*), C6(*S*), C8(*R*), C9(*S*), C10(*R*), C13(*S*), C14(*R*), C17(*S*), C20(*S*), C22(*R*).

Crystals of physalolactone [Figure 1] were obtained from methanol. A colorless crystal (0.40 × 0.35 × 0.20 mm) was analyzed at 120 K using a Nonius Kappa CCD diffractometer equipped with a Cyrostream 700 cryocooler. The analysis was conducted to a maximum 2θ value of 75.8° with monochromatized Mo Kα radiation; yielding 55055 reflections. The data were integrated and scaled using the HKL suite of programs consisting of XdisplayF, Denzo, and Scalepack. Unit cell parameters were retrieved and refined on 8,253 reflections with 1.0 < θ < 37.8°. The crystal data for **2**: C_28_H_39_ClO_8_⋅H_2_O, orthorhombic space group P2_1_2_1_2_1_, *a* = 10.9838(2) Å, *b* = 11.4987(2) Å, *c* = 22.2994(5) Å, *V* = 2816.4(1) Å^3^, *Z* = 4, *ρ* = 1.314 g/cm^3^, *μ* = 0.187 mm^−1^. The structure was solved using direct methods (SHELXD). All non-H atoms were refined anisotropically, while H-atoms were refined isotropically, with six methyl H atoms refined as riding in the final full-matrix least-squares refinement cycle on F, which converged at R_1_ = 0.053, wR_2_ = 0.051 and GOF = 1.105 for 7962 reflections with I > 3σ(I); a robust-resistant weighting scheme was used in the least-squares refinements of the 490 variables. The final Fourier difference map was featureless; the max./min. peaks were 0.68/–0.52 e^-^/Å^3^ and were located are chemically implausible positions. The absolute structure was deduced based on a Flack parameter of 0.01(6) refined using 6581 Friedel pairs. All crystallographic calculations were performed using Crystal Structure Analysis Package (4.2.2), Rigaku Corporation. Crystallographic data, including structure factor tables for the structure of compound **2**, have been deposited with the Cambridge Crystallographic Data Centre. To obtain copies of the data at no cost, you can apply to the Director at CCDC, 12 Union Road, Cambridge CB2 1EZ, UK (fax: +44-(0)1223-336033 or e-mail: deposit@ccdc.cam.ac.uk). The deposition number is: CCDC 1452313.

#### Acetylation reactions

Acetylation of compound **2** (10 mg) in pyridine (5 mL) and acetic anhydride (2 mL) was stirred for 24 h at room temperature (Frolow et al. 1981; Khan et al. 1999). Next, about 10 mL of deionized water were then added to the reaction solution and evaporated to dryness. The residue was purified by semi-preparative HPLC using a YMC PackPro C_18_ column (150 × 20 mm, 5 *µ*m). By employing a gradient of MeCN: H_2_O (30:70–80:20) at a flow rate of 1 mL/min, we successfully isolated compound **2a** (4.3 mg, 43% yield). ***Acetylation of Compound 3.*** A solution of **3** (5 mg) in pyridine (3 mL) and acetic anhydride (1 mL) was mixed and stirred for 24 hr at room temperature. Next, about 10 mL of deionized water was added to the reaction solution and evaporated to dryness. The residue was purified by semi-preparative HPLC (YMC PackPro C_18_ column, 150 × 20 mm, 5 *µ*m) and eluted with a gradient of MeCN: H_2_O (60:40–100:0) with a flow rate of 1.8 mL/min to yield compound **3a** (3.1 mg, 40% yield). ***Acetylation of Compound 4***. A solution of **4** (25 mg) in pyridine (10 mL) and acetic anhydride (2 mL) was stirred for 24 hr at room temperature. Next, about 10 mL of deionized water was added to the reaction solution and evaporated to dryness. The residue was purified by semi-preparative HPLC (YMC PackPro C_18_ column, 150 × 20 mm, 5 *µ*m) and eluted with a gradient of MeCN: H_2_O (60: 40–100: 0) with a flow rate of 2 mL/min, to yield compound **4a** (11.1 mg, 44.4% yield). ***Acetylation of Compound 5***. A solution of **5** (8 mg) in pyridine (5 mL) and acetic anhydride (2 mL) was stirred for 24 h at room temperature. Next, about 10 mL of deionized water was added to the reaction solution and evaporated to dryness. The mixture residue was purified by semi-preparative HPLC (YMC PackPro C_18_ column, 150 × 20 mm, 5 *µ*m) and eluted with a gradient of MeCN: H_2_O (10:90–50:50) with a flow rate of 1 mL/min to yield compound **5a** (2.7 mg, 33.75% yield). ***Acetylation of Compound 6***. A solution of **6** (10 mg) in pyridine (5 mL) and acetic anhydride (2 mL) was stirred for 24 h at room temperature. Next, about 10 mL of deionized water was added to the reaction solution and evaporated to dryness. The mixture residue was purified by semi-preparative HPLC (YMC PackPro C_18_ column, 150 × 20 mm, 5 *µ*M) and eluted with a gradient of MeCN: H_2_O (50:50–100:0) with a flow rate of 1.2 mL/min to yield compound **6a** (6.8 mg, 68% yield).

### Inhibition of tumor necrosis factor-α activated nuclear factor-kappa B

To study the inhibition of tumor necrosis factor-alpha (TNF-α)-induced activation of nuclear factor-kappa B (NF-κB), human embryonic kidney 293 cells (HEK293) stably transfected with an NF-κB-responsive luciferase reporter construct (Cat. No. RC0014, Panomics, Fremont, CA, USA) were utilized, following previously described protocols (Kondratyuk et al. 2012). This cell line incorporates a chromosomally integrated luciferase reporter, which produces luciferase enzyme upon NF-κB activation. The enzyme reacts with its substrate to produce a luminescent signal, detectable *via* luminometry. Cells were seeded at a density of 2 × 10^4^ cells/mL in 96-well plates and cultured in Dulbecco’s Modified Eagle Medium (DMEM, Cat. No. D6429, Sigma-Aldrich, Saint Louis, MO, USA) supplemented with 10% fetal bovine serum (FBS, Cat. No. A5670701, Gibco^™^, Thermo Fisher Scientific, Waltham, MA, USA). The medium also contained 100 µg/mL streptomycin sulfate and 100 IU/mL penicillin G (Cat. No.15140 Gibco BRL, Grand Island, NY, USA). The cells were then treated with varying concentrations of the test sample and stimulated with recombinant human TNF-α (2 ng/mL, Cat. No. HZ-1014, Proteintech Group, Inc. Rosemont, IL, USA).

Following a 6-hour incubation, the cells were rinsed with phosphate buffer saline (PBS), lysed with 40 μL of 1X Reporter Lysis Buffer (Cat. No. E2661, Promega, Madison, WI, USA), and subjected to a freeze-thaw cycle (−80 °C/37 °C). Luminescence, generated by the luciferase reaction, was quantified using the Luc assay system (Cat. No. N1610, Promega, Madison, WI, USA) and a LUMIstar Galaxy luminometer (BMG, Offenburg, Germany). Samples exhibiting over 70% inhibition at 50 µM were further analyzed to determine their IC_50_ values. The positive control, *N*_α_-tosyl-l-phenylalanine chloromethyl ketone (TPCK, Cat. No. T4376, Sigma-Aldrich, Saint Louis, MO, USA), demonstrated an IC_50_ of 5.09 µM.

### Inhibition of nitric oxide production in lipopolysaccharide-activated murine macrophage RAW 264.7 cells

The inhibition of nitric oxide (NO) production in lipopolysaccharide (LPS)-stimulated murine macrophage RAW 264.7 cells was evaluated following previously described methods (Min et al. 2009). RAW 264.7 cell line was purchased from ATCC (Cat. No. TIB-71, Manassas, VA, USA) and cultured in Dulbecco’s modified Eagle’s medium (DMEM, Cat. No. 12800-082, Invitrogen, Massachusetts, USA) supplemented with 10% heat-inactivated fetal bovine serum (FBS, Cat. No. 26140, Gibco BRL Co, Grand Island, NY, USA), 100× penicillin G and streptomycin (Cat. No.11074440001, Sigma-Aldrich, Saint Louis, MO, USA) and 2 mM l-glutamine (Cat. No. 25030, Gibco BRL Co, Grand Island, NY, USA). Cells were seeded at 10 × 10^5^ cells per well in 96-well plates and incubated for 24 h at 37 °C in a humidified atmosphere containing 5% CO_2_.

Test compounds were dissolved in phenol red-free DMEM (Cat. No. 12-917 F, Lonza, Massachusetts, USA) and added to the cells as a pretreatment for 30 min. The cells were then exposed to LPS (1 µg/mL, Cat. No. L-2762, Sigma-Aldrich, Saint Louis, MO, USA) for 20 h. Nitric oxide levels were quantified using the Griess reagent, which consists of a 1:1 (v/v) mixture of 1% sulfanilamide (Cat.No. A13001, Alfa Aesar, Massachusetts, USA) in H_3_PO_4_ and *N*-(1-naphthyl)ethylenediamine dihydrochloride (Cat. No. 102397, MP Biomedicals, USA). A standard curve was generated using sodium nitrite (Cat. No. 44227, Alfa Aesar, Massachusetts, USA), and IC_50_ values were calculated using Table Curve 2D software (Version 4.07, AISN Software Inc., Richmond, VA, USA). Also, a sulforhodamine B (SRB, Cat. No. S9012, Sigma-Aldrich, Saint Louis, MO, USA) assay was performed to assess the cytotoxic effects of the test compounds on RAW 264.7 cells. l-*N*^G^-Monomethyl arginine citrate (l-NMMA, Cat. No. 10005031, Cayman Chemical, Ann Arbor, MI, USA), served as the positive control for this assay, with an IC_50_ value of 23.5 µM.

## Results and discussion

**Figure 2.**
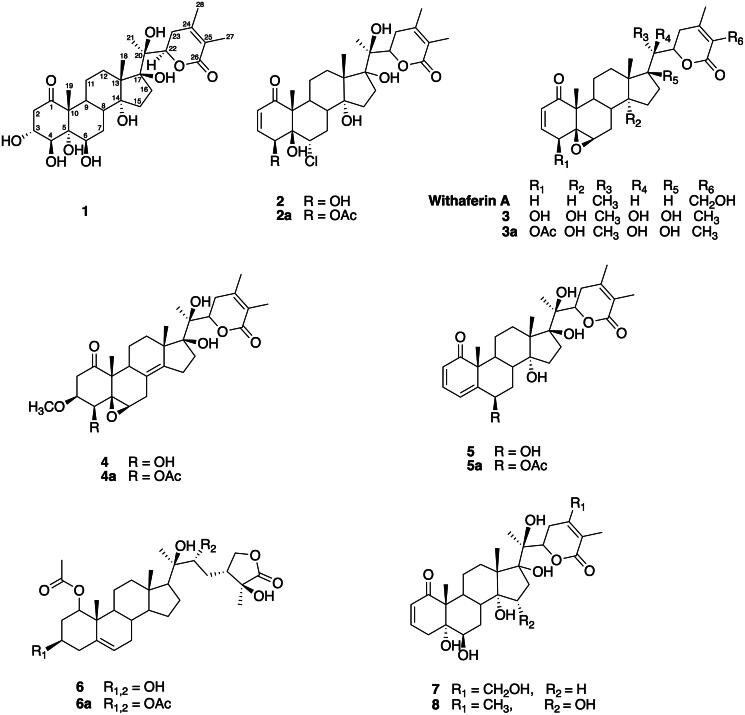
Chemical structures of compounds **1-8** and their acetylated derivatives **2a-6a**.

The ethanolic extract of fresh *P. peruviana* fruits was suspended in deionized water and successively partitioned with *n*-hexane, EtOAc, and *n*-butanol. The *n*-butanol soluble fraction was first applied to Sephadex LH-20 and further separated with several column chromatographic procedures (Sang-Ngern et al. 2016). This isolation procedure, with bioassay-guided fractionation, resulted in a new compound, physaperuvin K [**1,** (20*S*,22*R*)-3α,4β,5α,6β,14α,17β,20β-heptahydroxy-1-oxowitha-24-ene-26,22-olide]. In addition, seven known compounds were isolated from the crude extracts of aerial parts of *P. peruviana*, i.e., physalolactone (**2**) (Ray et al. 1978; Ali et al. 1984), 4β-hydroxywithanolide E (Dinan et al. 1997) (**3**), physalactone (Maslennikova et al. 1977) (**4**), withaperuvin C (Sahai et al. 1982) (**5**), perulactone C (Fang et al. 2009) (**6**), phyperunolide B (Lan et al. 2009) (**7**), and coagulin H (Kirson et al. 1971) (**8**). Five acetylated derivatives (Khan et al. 1999; Fang et al. 2012) (**2a–6a**) were prepared using parent compounds **2–6**. The identification and structure elucidation of compounds **1** and **2** and the bioactivities of compounds **1–8** and acetylated derivatives **2a–6a** are presented herein [Figure 2].

Compound **1** was isolated as a yellowish solid. Its HRESIMS ([2M + K]^+^ *m/z* 1115.5175, C_56_H_84_O_20_ calcd 1115.5193) along with its NMR spectroscopic data corresponding to a molecular formula of C_28_H_42_O_10_. The IR spectrum displayed the presence of a hydroxyl (3335 cm^−1^), and conjugated ketone (1682 cm^−1^) functionalities. Analysis of its ^1^H NMR spectra data (Table 1) revealed the presence of signals for five tertiary methyls [*δ*_H_ 1.09, 1.24, 1.37, 1.84, and 1.96], and three olefinic protons [*δ*_H_ 4.23 (1H, m), 4.29 (1H, d, *J* = 6.4 Hz), 4.08 (1H, dd, *J* = 6.9, 2.1 Hz)]. The ^13^C NMR spectrum of **1** showed 28 resonances, comprised of five methyls [*δ*_C_ 12.5 (C-27), 15.5 (C-19), 20.7 (C-18), 21.0 (C-28) and 80.0 (C-20)], seven methylenes [*δ*_C_ 22.1 (C-11), 27.1 (C-7), 31.6 (C-12), 33.2 (C-15), 34.6 (C-23), 37.7 (C-16) and 42.8 (C-2)], six methines [*δ*_C_ 35.8 (C-8), 35.3 (C-9) and 83.2 (C-22)], including three oxygenated [*δ*_C_ 74.8 (C-3), 78.3 (C-4) and 78.8 (C-6)], and ten quaternary carbons (including one ketone carbonyl) [*δ*_C_ 213.4 (C-1), an ester carbonyl [*δ*_C_ 169.3 (C-26)], four olefins [*δ*_C_ 55.9 (C-13), 56.0 (C-10), 122.1 (C-25) and 153.5 (C-24)], and four oxygenated [*δ*_C_ 77.5 (C-5), 80.0 (C-20), 83.8 (C-14), and 88.8 (C-17)]. The remaining seven hydrogen atoms were assigned as OH protons [including four oxygenated quaternary carbons at *δ*_C_ 77.5 (C-5), 83.8 (C-14), 88.8 (C-17), and 80.0 (C-20)].

**Figure 3.**
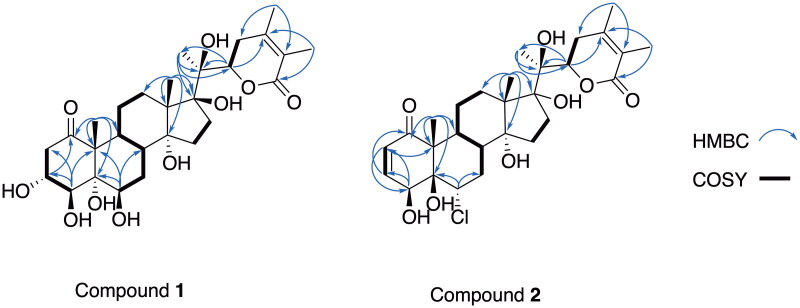
HMBC correlations for compounds **1** and **2**.

**Figure 4.**
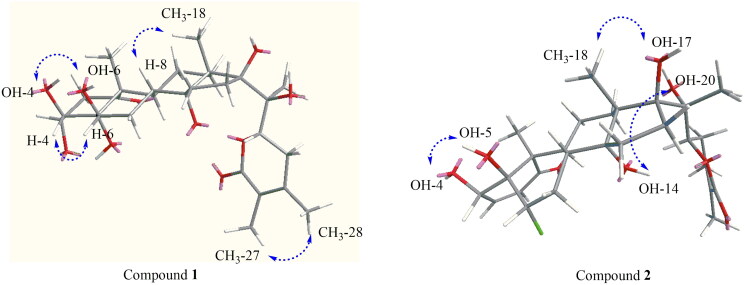
Key NOESY correlation for compounds **1** and **2**.

The spectroscopic data of **1** were closely related to a withaperuvin, which was previously isolated from the same plant (Frolow et al. 1981; Rahman et al. 1998). However, the differences in the resonance peak of ring A. Notably, the olefinic protons at the C-2-C-3 bridge in withaperuvin were absent in compound **1**. Furthermore, compound **1** contains OH groups at the C-3 and C-4 positions, along with two methylene protons at *δ*_H_ 2.39 (2H, dd, *J* = 18.0, 3.5 Hz) at C-2. Further confirmation of this supposition was provided in the HMBC spectrum (Figure 3). The H-22 oxymethine proton, resonating at *δ*_H_ 4.81 (1H, dd, *J* = 13.2, 3.9 Hz), exhibited both axial-axial and axial-equatorial relationships with the C-23 protons. This observation was consistent with the C-22 *R* configuration (Frolow et al. 1981; Khan et al. 1999). Additionally, the circular dichroism (CD) data for this compound showed a positive Cotton effect at 250 nm (Δɛ = +865.25) (Kirson et al. 1971), further supporting this configuration. The relative configurations of **1** were assigned from NOESY spectroscopy data (Figure S6, Supporting Information). The NOE correlations of CH_3_-18 (*δ*_H_ 1.09) with H-8 indicated that CH_3_-18 and H-8 were located on the same side of the molecule and arbitrarily assigned as the *β-*orientation. Furthermore, the NOE correlation observed for compound **1** between H-4 and H-6, along with the absence of a correlation between H-6 and H-8, suggests that H-4 and H-6 are oriented in the *α*-forms (see Figure 4 and Figure S7 in the Supporting Information). Hence, the structure of compound **1** was identified, and the new compound, (20*S*,22*R*)-3α,4β,5α,6β,14α,17β,20β-heptahydroxy-1-oxowitha-24-ene-26,22-olide. We had previously isolated and reported physaperuvins G, I, and J as new compounds. Thus, compound **1**, isolated from *Physalis peruviana*, was given the trivial name physaperuvin K.

Compound **2** was isolated as colorless needles. Physalolactone was previously reported by Ray et al. in 1978, yet its structure was proposed based on 90 MHz proton NMR and chemical derivatives. Its molecular formula was established as C_28_H_39_ClO_8_ based on its HRESIMS (*m/z* 561.2224; calcd for C_28_H_39_ClNaO_8_, 561.2231). The presence of two [M + H]^+^ peaks in a ratio of 3:1 to the expected [M + H]^+^ for an unchlorinated ion peak, indicated the presence of one chlorine in the molecule. The IR spectrum showed absorptions attributed to carbonyl (1675 cm^−1^) and hydroxyl (3311 cm^−1^) functionalities, and a strong UV absorption at λ max 213 nm suggests the presence of an α,β-unsaturated lactone. The ^13^C NMR and DEPT data (Table 1) showed 28 carbon resonances assignable to ten quaternary carbons (including one keto carbonyl) at δ_C_ 203.6 and an ester carbonyl at δ_C_ 168.9, four olefins at δ_C_ 55.6, 58.1, 153.2, 121.8, and four oxygenated at δ_C_ 79.5, 79.8, 88.0, 83.2, five methyls at δ_C_ 9.9, 12.5, 19.3, 20.9, 21.2, six methylenes at *δ*_C_ 23.6, 31.0, 32.9, 35.3, 37.2, 39.6, five methines at δ_C_ 35.5, 39.9, 65.7, 65.8, 82.2, and two olefinic carbons at δ_C_ 126.9, 147.3. The NMR spectroscopic data of **2** (Table 1) also closely resembled those of a withanolide skeleton. The five methyl moieties resonated at δ_H_ 1.04 (3H, s), 1.21 (3H, s), 1.36 (3H, s), 1.81 (3H, s), and 1.92 (3H, s) corresponding to the ­quaternary methyls C-18, 19, 21, 27 and 28, respectively, of a withanolide derivative. The NMR data of **2** were comparable to those of two other chlorinated withanolides: 6α-chloro-5β-hydroxywithaferin A (Kirson and Glotter 1981) and 6α-chloro-5β,17α-dihydroxywithaferin A (Tong et al. 2011). The main differences between **2** and 6α-chloro-5β-hydroxywithaferin A are as follows: Compound **2** contains a quaternary carbon (δ_C_ 88.0), while 6α-chloro-5β-hydroxywithaferin A contains a methine carbon (δ_C_ 51.3 C-17); a quaternary carbon (δ_C_ 83.2) in **2** and a methine carbon (δ_C_ 55.3 C-14) in the latter; and a tertiary methyl (δ_H_ 1.36 s; δ_C_ 19.3) in **2** and a secondary methyl (δ_H_ 0.95 (d); δ_C_ 13.3) in the latter. The HMBC correlations are between CH_3_-21 (δ_H_ 1.36 s; δ_C_ 19.3) and C-17 (δ_C_ 88.0), and between CH_3_-18 (δ_H_ 1.04 s; δ_C_ 21.2) and C-17 (δ_C_ 88.0) in **2**. The final assignment of NMR data (Table 1) was completed using 2D NMR, including ^1^H-^1^H COSY, HSQC, and HMBC spectra.

The NOESY correlations of compound **2** were recorded in DMSO-*d*_6_. The correlations of CH_3_-18 (δ_H_ 0.92)/OH-17 (δ_H_ 4.73), and CH_3_-19 (δ_H_ 1.08)/H-6 (δ_H_ 4.35), suggest that H-6 and OH-17 are oriented in the *β* configuration (Figure S14, Supporting information). Furthermore, the observed NOESY correlations between OH-14 (δ_H_ 5.79) and OH-20 (δ_H_ 6.68) indicate that these groups are cofacial and support the conclusion that they possess *α*-orientations, consistent with the single-crystal X-ray diffraction analysis of compound **2** (Figure 1).

Additionally, the OH-17 and Me-18 groups are oriented in the *β* form. The resonance of the H-22 oxymethine proton at δ_H_ 4.76 (1H, dd, *J* = 13.2, 3.8 Hz) demonstrates both axial-axial and axial-equatorial relationship with the C-23 protons, consistent with the *R* configuration of C-22 (Frolow et al. 1981). This conclusion is further supported by the CD data showing a Cotton effect at 236 nm (Δɛ = +992.81) (Kirson et al. 1971) and, more importantly, the X-ray structure. Moreover, the X-ray structure of physalolactone confirms that the absolute configuration at the C-20 stereogenic center is *S*; see Figure 1. Thus, based on all the evidence presented, compound **2** was established as (20*S,*22*R*)-4β,5β,14α,17β,20α-pentahydroxy-6α-chloro-1-oxowitha-2,24-diene-22,26-olide, or physalolactone.

Naturally occurring halogenated steroids are rare metabolites produced by higher plants (Gribble 1994). Chlorinated withanolides, in particular, are not commonly found in the Solanaceae family (Tschesche et al. 1968; Nittala et al. 1981; Dembitsky and Tolstikov 2003). For example, chlorinated withanolides have been isolated from several species, including *Jaborosa integrifolia* Lam. (Tschesche et al. 1968) (jaborosalactones C and E), *Withania frutescens* (L.) Pauquy (Gonzalez et al. 1974) (6*α*-chloro-5*β*-hydroxywithaferin A), and *W. somnifera* (L.) Dunal, which yields compounds such as 6*α*-chloro-5*β*-hydroxywithaferin D, 4-deoxyphysalactone; (Dembitsky and Tolstikov 2003), withanolide C; (Bessalle and Lavie 1992), withanolide Z; (Pramanick et al. 2008), and 27-acetoxy-5*β*-chloro-6α-hydroxywithaferine A (Sang-Ngern et al. 2016). Additional chlorinated withanolides, such as 6*α*-chloro-5*β*,17*α*,-dihydroxywithaferin A, and 6*α*-chloro-5*β*-hydroxywithaferin A have been identified as well (Tong et al. 2011). Notably, chlorinated withanolides have also been isolated from *P. peruviana*: physalolactone and 4-deoxyphysalolactone; (Ray et al. 1978; Frolow et al. 1981), as well as phyperunolide C and 5*α*-chloro-6*β*-hydroxywithanolide E, along with its isomer, 6*α*-chloro-5*β*-hydroxywithanolide E (Nittala et al. 1981). However, very few studies have examined the biological activities of the chlorinated withanolides obtained from *P. peruviana* (Chang et al. 2016; San-Ngern et al. 2016; Park et al. 2019).

A previous study suggested that chlorinated withanolides isolated from *Jaborosa magellanica* (Griseb.) Dusén, *P. peruviana, W. frutescens* and *W. somnifera* might be artifacts of chlorination, potentially resulting from the CHCl_3_/MeOH solvent systems and reaction with trace amounts of HCl used during the extraction process (Fajardo et al. 1991). The debate over whether chlorinated withanolides are naturally occurring or the result of artifacts remains somewhat unresolved. However, Nittala et al. 1981 have advocated that chlorinated withanolides are naturally occurring rather than artifacts. They proposed that the chlorine found in 4-deoxyphysalolactone, from *W. somnifera* (L.) Dunal and *Acnistus breviflorus* Sendtn. (Nittala et al. 1981; Fajardo et al. 1991) originates from the substantial amounts of NaCl present in the soil where these plants grow. This hypothesis aligns with the fact that many terrestrial plants possess chloroperoxidase enzymes capable of chlorinating compounds in the presence of chloride ions. In research conducted by Nittatla et al. the hydrochlorination of withanolide D using 32% HCl resulted in the formation of 6*α*-chloro-4*β*,5*β*-20*α*-trihydroxy-1-oxo-22*R*-withanolide. This finding reinforces the hypothesis that the C-5/C-6 epoxide rings have undergone a diaxial opening, producing a 6,5-chlorohydrin with an inverted configuration (Nittala et al. 1981). These *trans*-6,5-chlorohydrins are also produced alongside their proposed biosynthetic precursors, the 5,6-epoxides, as reported by other research teams (Frolow et al. 1981). Carr et al. (1996) were the first to show the formation of carbon-chlorine bonds in the chlorohydrin intermediates, specifically (5*α*-hydroxy-6*β*-chloro)- and (5*α*-chloro-6*β*-hydroxy)-cholesterols.

Turning to the biological activities of these compounds, nitric oxide (NO) is a reactive nitrogen species that plays crucial roles in various cell metabolism and development processes. Due to its involvement in many physiological and pathological functions, abnormal or excessive NO production can lead to diseases. Nitric oxide synthase (NOS) produces nitric oxide from l-arginine. There are three main isoforms of NOS: endothelial NOS (eNOS), neuronal NOS (nNOS), and inducible NOS (iNOS). The iNOS gene is consistently associated with chronic inflammation, tumor production (Korhonen et al. 2005; Nomelini et al. 2008), and metastasis (Hickok and Thomas 2010). Furthermore, the overproduction of NO modulates various cancer-related events including angiogenesis, apoptosis, cell cycle, invasion, and metastasis (Choudhari et al. 2013). Accordingly, we tested the potential anti-inflammatory effect of withanolides **1–8** and their acetate derivatives (**2a–6a**) by assessing their ability to inhibit NO-production with LPS-treated murine macrophage RAW 264.7 cells. LPS induces the production of pro-inflammatory cytokines (Nicholas et al. 2007) and was used to stimulate NO production.

The results of the NO assay (Table 2) indicate inhibitory effects of 4β*-*hydroxywithanolide E (**3**) and its acetate derivative (**3a**), with IC_50_ values of 0.32 and 0.24 μM, respectively. However, these compounds also exhibited cytotoxicity at higher concentrations, with cytotoxic IC_50_ values of 14 and 2.1 μM, respectively. On the other hand, compounds **1**, **2**, **2a**, **4**, **5**, **5a**, and **6** showed inhibition of NO-production without significant cytotoxicity. For instance, physalolactone (**2**), physalactone (**4**), withaperuvin C (**5**), and withaperuvin C acetate (**5a**) displayed moderate inhibitory activities with IC_50_ values ranging from 1.8 to 13.3 μM, but only modest cytotoxicity at a concentration of 50 μM. Notably, physalolactone (**2**) yielded an IC_50_ value of 3.3 μM without causing cytotoxic effects. Overall, the potency of withanolides was greater than that of l-*N*^G^-monomethyl arginine citrate, the positive control used for this assay (IC_50_ 23.5 ± 3.4 μM) (Table 2).

**Table 2.** Anti-inflammatory activities of compounds **1–8** and their derivatives from *P. peruviana.*

Compounds	NF-κB assay			Nitrite assay		
	% inhib.^a^	IC_50_ (μM)	% surv.^b^	% inhib.^c^	IC_50_ (μM)	% surv.^d^
**1**	93.4 ± 1.2	0.01 ± 0.01	100 ± 24.5	82.4 ± 1.1	13.3 ± 0.2	100 ± 1.1
**2**	97.4 ± 1.1	0.06 ± 0.02	100.0 ± 14.4	99.4 ± 0.1	3.3 ± 0.3	96.1 ± 3.2
**2a**	92.2 ± 0.6	1.10 ± 0.42	12.3 ± 7.58	99.2 ± 2.6	8.7 ± 0.6	93.2 ± 2.3
**3**	99.5 ± 0.1	0.04 ± 0.03	88.6 ± 10.1	99.2 ± 0.4	0.32 ± 0.02	50.1 ± 2.3
**3a**	99.9 ± 0.9	0.47 ± 0.61	2.2 ± 2.6	99.2 ± 0.4	0.24 ± 0.01	38.9 ± 2.3
**4**	66.7 ± 3.9	11.0 ± 2.3	100.0 ± 16.6	97.9 ± 2.0	1.8 ± 0.17	90.7 ± 5.0
**4a**	88.0 ± 1.3	0.33 ± 0.1	77.7 ± 5.3	99.8 ± 0.3	0.24 ± 0.01	40.3 ± 4.1
**5**	85.0 ± 4.4	5.6 ± 2.1	100.0 ± 8.0	99.2 ± 0.7	2.4 ± 0.2	79.3 ± 1.2
**5a**	78.3 ± 3.0	2.4 ± 1.7	47.0 ± 6.0	99.0 ± 0.3	1.8 ± 0.1	98.8 ± 1.4
**6**	56.1 ± 3.9	1.1 ± 0.48	36.5 ± 1.9	84.2 ± 3.7	11.8 ± 0.3	95.3 ± 11.0
**6a**	77.4 ± 7.3	10.5 ± 2.55	21.8 ± 0.8	20.6 ± 3.0	nd	92.0 ± 5.0
**7**	41.0 ± 6.9	nd	81.6 ± 8.4	7.4 ± 3.5	nd	100.0 ± 14.1
**8**	70.2 ± 8.1	8.9 ± 1.1	82.2 ± 3.9	67.1 ± 2.2	16.7 ± 0.4	100.0 ± 6.3
l-NMMA^e^					23.5 ± 3.4	
TPCK^f^		7.08 ± 1.72				

^a^% inhibition at a concentration of 50 μM, ^b^ % survival at a concentration of 50 μM, ^c^ % inhibition of NO at 50 μM, ^d ^% survival at a concentration of 50 μM, ^e^l-*N*^G^-Monomethyl arginine citrate, positive control for NO inhibition, ^f^*N*_α_-Tosyl-l-phenylalanine chloromethyl ketone, positive control for NF-κB inhibition.

Mechanisms of withanolide-mediated inhibition of NO-production have been suggested. For example, 4β-hydroxywithanolide E (**3**) inhibited NO-production in LPS-stimulated RAW 264.7 cells with an IC_50_ value of 0.9 µM while simultaneously reducing the mRNA and protein expression of iNOS and cyclooxygenase (COX)-2. Furthermore, 4β-hydroxywithanolide E (**3**), isolated from *P. peruviana*, mediated anti-inflammatory effects through the unexpected mechanism of inhibiting the transcription of iNOS and COX-2 *via* protein kinase B (Akt) and signal transducer and activator of transcription 1 (STAT1)-related signaling pathways (Choudhari et al. 2013; Park et al. 2019). Therefore, withanolides may have a unique potential for cancer chemoprevention due to their anti-inflammatory properties.

The anticancer properties of withaferin A [4β,5β,6β,22*R*)-4,27-dihydroxy-5,6-22,26-diepoxyergosta-2,24-diene-1,26-dione; Figure 4], commonly known as Ashwagandha, Indian ginseng or Indian winter cherry, have been intensively studied. This material, currently marketed as a dietary supplement (Xing et al. 2023), functions through a variety of mechanisms (Lee and Choi 2016), including inhibition of NF-κB. As reported by Ichikawa et al. (2006), withaferin A and its acetate derivative, inhibit TNF-α-induced NF-κB activation through inhibition of the master regulator of the NF-κB, i.e., IKK (inhibitor of nuclear factor-κB kinase), which leads to suppression of phosphorylation and degradation of IκBα. Possibly by this same mechanism, all of the withanolides currently reported inhibited TNF-α-induced NF-κB activation, with varying levels of potency (Table 2). Particularly notable are compounds **1**, **2,** and **3**, with IC_50_ values of 10, 60, and 40 nM. Individually, each of these three compounds has unique structural features, so it is difficult to attribute the inhibitory activity to any particular functional group. In fact, a comparison of **2** with **5** would tend to diminish emphasis on the α,β-unsaturated lactone, a comparison of **1** and **3** with **2** would tend to diminish emphasis on chlorination, and a comparison of **1** and **2** with **3** would tend to diminish emphasis of epoxidation. Also, acetylation did not reveal critical structural elements. The activity of **6a** was diminished by about 10-fold relative to **6**, whereas the activity of **4a** was enhanced by over 30-fold relative to **4**. It will be of interest to further explore the mode of action of these compounds, especially **1** since it is found in a consumable part of the plant.

Pro-inflammatory cytokines [e.g., interleukin (IL)-6, IL-8, IL-10, TNF-α, matrix metallopeptidase (MMP)-9 and tissue inhibitor of metalloproteinases (TIMP)-1] are induced by LPS through a variety of mechanisms (Liu et al. 2018), and inhibition of this response is anticipated to reduce inflammation, tumor formation (Korhonen et al. 2005; Nomelini et al. 2008) and metastasis (Hickok and Thomas 2010). Here, we monitored the potential of the isolates to inhibit NO production with cultured murine macrophage RAW 264.7 cells (Table 2). An anticipated mechanism of inhibition, i.e., reduced production of iNOS, includes interference with the NF-κB pathway. If this were the case, however, a good correlation with the activity observed with both the NO and NF-κB assays would be expected. A strong correlation of the potency of the individual compounds was not observed when comparing these two assays. Thus, the mechanism of NO inhibition is uncertain, but it is clear that some of the isolates were very effective in this capacity. Inhibitory activity was generally in the low micromolar range, and every compound tested was more active than the positive control of the assay, l-NMMA. The most active compounds were **3a** and **4a**, possibly indicating that epoxide functionality enhances activity. It is also of interest that these two compounds were among the most active in the NF-κB assay, possibly supporting a mechanistic interrelationship in these compounds. In any case, inhibition of pro-inflammatory activity by these withanolides, in the absence of strong cytotoxic responses, is promising. It would be of value to assess the full anti-inflammatory potential of these substances.

## Conclusions

In the current study, a new compound, namely physaperuvin K (**1**), was discovered from the edible fruit of *P. peruviana*. In addition, physalolactone (**2**), a rare chlorinated withanolide, was isolated from aerial parts of *P. peruviana*, along with six known compounds: 4β-hydroxywithanolide E (Dinan et al. 1997) (**3**), physalactone (Maslennikova et al. 1977) (**4**), withaperuvin C (Sahai et al. 1982) (**5**), perulactone C (Fang et al. 2009) (**6**), phyperunolide B (Lan et al. 2009) (**7**), and coagulin H (Kirson and Glotter 1981) (**8**). An assessment of the biological potential showed significant anti-inflammatory and chemopreventative effects at nanomolar concentrations, without overt cytotoxicity. This highlights the value of this traditional medicinal plant, which has been used traditionally across various cultures and ethnicities. Its effectiveness is supported by the biological activity of its phytoconstituents.

## Supplementary Material

Supporting Information 121924.docx
