# Supplementary material for: Biologically active withanolides from Physalis peruviana
Source: Pharm Biol. 2025 Apr 26;63(1):334–43. doi: 10.1080/13880209.2025.2488136 (PMC12035931; doi:10.1080/13880209.2025.2488136)
Supplement: Supporting Information 121924.docx [file IPHB_A_2488136_SM1535.docx]

**SUPPLEMENTARY MATERIALS**

**Biologically active withanolides from *Physalis peruviana***

Mayuramas Sang-Ngern,^a,b^ Ashley Fukuchi,^a^ Tamara P. Kondratyuk,^a^ Eun-Jung Park,^a,c^ Charles J. Simmons, ^d^ Marisa M. Wall,^e^  Sam E. Lorch, ^†^ John M. Pezzuto,^a,c^ and Leng Chee Chang^a,^*

*^a^Department of Pharmaceutical Sciences, The Daniel K. Inouye College of Pharmacy, University of Hawai‘i at Hilo, Hilo, Hawaii, 96720, United States;*

*^b^School of Cosmetic Science, Mae Fah Luang University, Tasud, Muang District, Chiang Rai, 57100, Thailand;*

*^c^College of Pharmacy and Health Sciences, Western New England University, Springfield, Massachusetts, 01119, United States;*

*^d^X-ray Diffraction Laboratory, Department of Chemistry, 200 West Kawili Street, University of Hawai‘i at Hilo, Hilo, Hawai‘i, 96720, United States;*

*^e^Daniel K. Inouye U.S. Pacific Basin Agricultural Research Center, Hilo, Hawai‘i, 96720, United States*

*†Lani ko Honua Berry Farm, Pepeekeo, Hawai‘i, 96783, United States. This work would not have been possible without the support, enthusiasm, and camaraderie of Sam E. Lorch, who regretfully succumbed to the consequences of cancer during the investigation. The authors respectfully dedicate this paper in memory of him.*

* **Corresponding Author:** Leng Chee Chang, Ph.D.

The Daniel K. Inouye College of Pharmacy, 200 W. Kawili Street, University of Hawai‘i at Hilo, Hilo, HI 96720, USA. Tel: +1-808-932-8124, Fax: +1-808-932-8117;

E-mail address: [lengchee@hawaii.edu](mailto:lengchee@hawaii.edu)

| **Table of Contents** | | **Page** |
| --- | --- | --- |
| **Figure S1.** | Chemical structures of compounds **1** and **2** isolated from *Physalis peruviana* | 3 |
| **Figure S2**. | ^1^H NMR spectrum (400 MHz, CD_3_OD) of compound **1**. | 4 |
| **Figure S3**. | ^13^C NMR spectrum (100 MHz, CD_3_OD) of compound **1**. | 5 |
| **Figure S4**. | COSY NMR spectrum (400 MHz, CD_3_OD) of compound **1**. | 6 |
| **Figure S5**. | HSQC NMR spectrum (400 MHz, CD_3_OD) of compound **1**. | 7 |
| **Figure S6**. | HMBC NMR spectrum (400 MHz, CD_3_OD) of compound **1**. | 8 |
| **Figure S7**. | NOESY NMR spectrum (400 MHz, CD_3_OD) of compound **1**. | 9 |
| **Figure S8**. | ^1^H NMR spectrum (400 MHz, CD_3_OD + 20% CDCl_3_) of compound **2**. | 10 |
| **Figure S9**. | ^13^C NMR spectrum (100 MHz, CD_3_OD + 20% CDCl_3_) of compound **2**. | 11 |
| **Figure S10**. | COSY NMR spectrum (400 MHz, CD_3_OD + 20% CDCl_3_) of compound **2**. | 12 |
| **Figure S11**. | HSQC NMR spectrum (400 MHz, CD_3_OD + 20% CDCl_3_) of compound **2.** | 13 |
| **Figure S12**. | HMBC NMR spectrum (400 MHz, CD_3_OD + 20% CDCl_3_) of compound **2.** | 14 |
| **Figure S13**. | NOESY NMR spectrum (400 MHz, CD_3_OD + 20% CDCl_3_) of compound **2**. | 15 |
| **Figure S14**. | ^1^H NMR spectrum (400 MHz, DMSO) of compound **2**. | 16 |
| **Figure S15**. | NOESY NMR spectrum (400 MHz, DMSO) of compound **2**. | 17 |
| **Figure S16**. | HR-ESIMS of compound **1**. | 18 |
| **Figure S17**. | HR-ESIMS of compound **2**. | 18 |

**Figure S1.** Chemical structures of compounds **1**and **2** isolated from *Physalis peruviana*


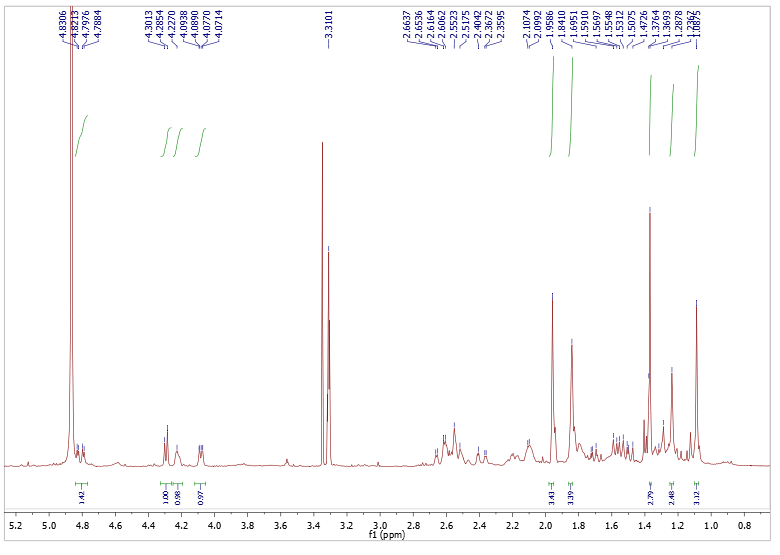


| **Figure S2**. | ^1^H NMR spectrum (400 MHz, CD_3_OD) of compound **1**. |
| --- | --- |


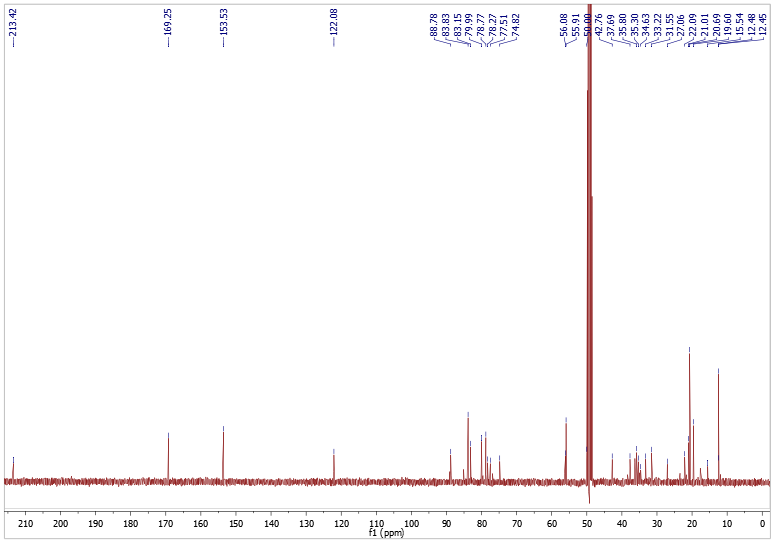


| **Figure S3**. | ^13^C NMR spectrum (100 MHz, CD_3_OD) of compound **1.** |  |
| --- | --- | --- |


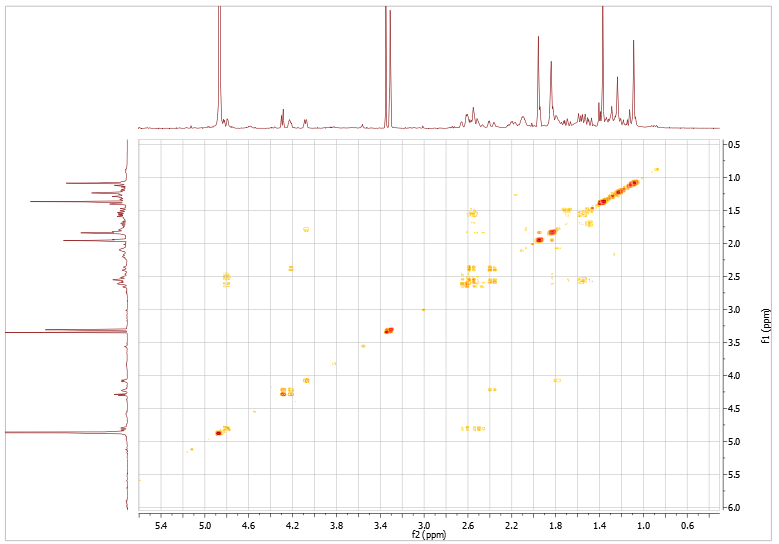


| **Figure S4**. | COSY NMR spectrum (400 MHz, CD_3_OD) of compound **1**. |
| --- | --- |


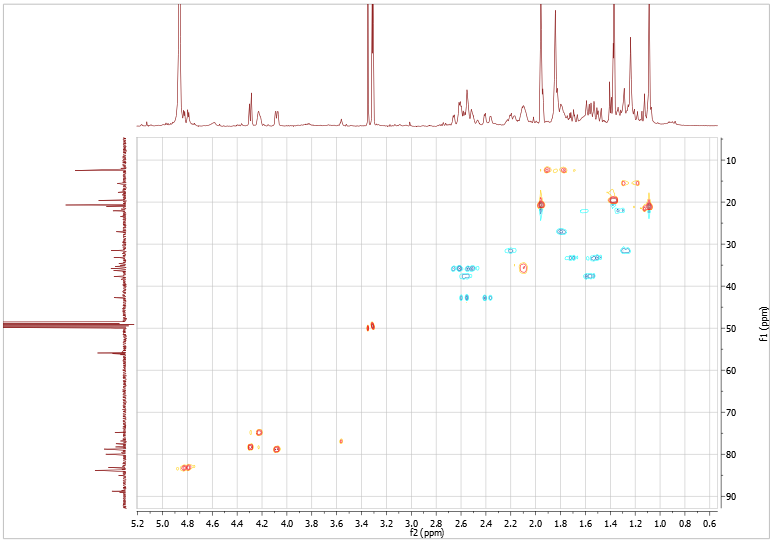


| **Figure S5**. | HSQC NMR spectrum (400 MHz, CD_3_OD) of compound **1**. |  |
| --- | --- | --- |

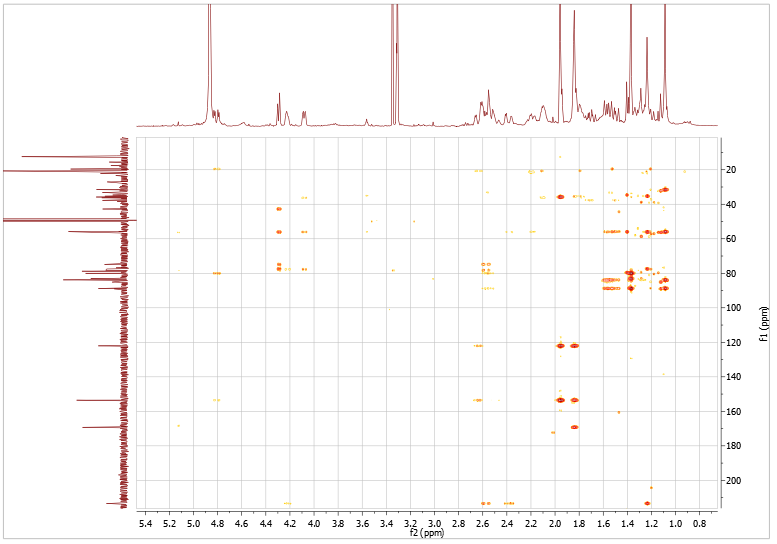


**Figure S6**. HMBC NMR spectrum (400 MHz, CD_3_OD) of compound **1.**

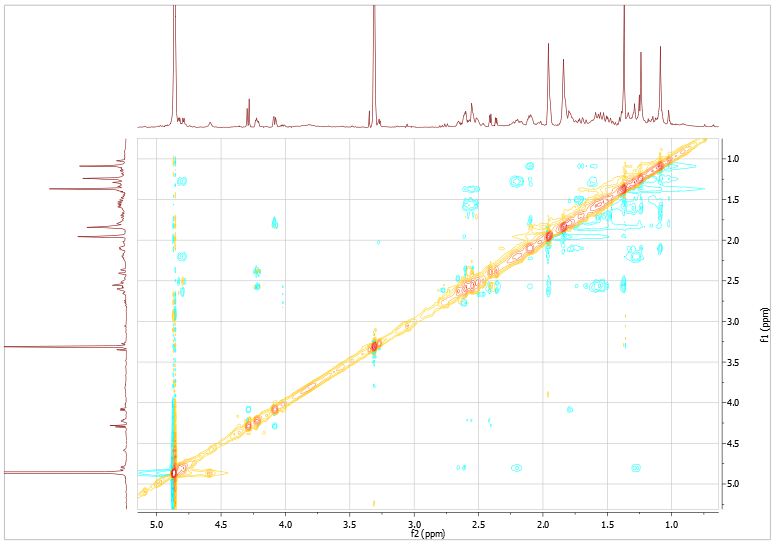


**Figure S7**. NOESY NMR spectrum (400 MHz, CD_3_OD) of compound **1**.


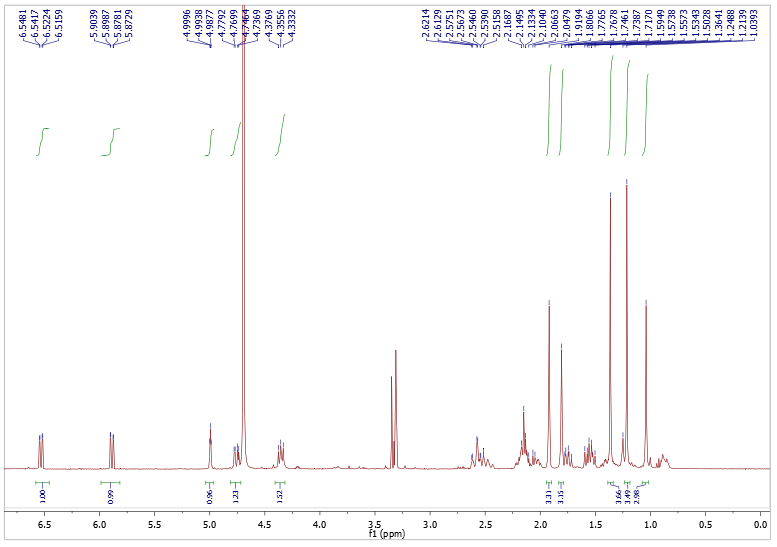
**Figure S8**. ^1^H NMR spectrum (400 MHz, CD_3_OD + 20% CDCl_3_) of compound **2**.

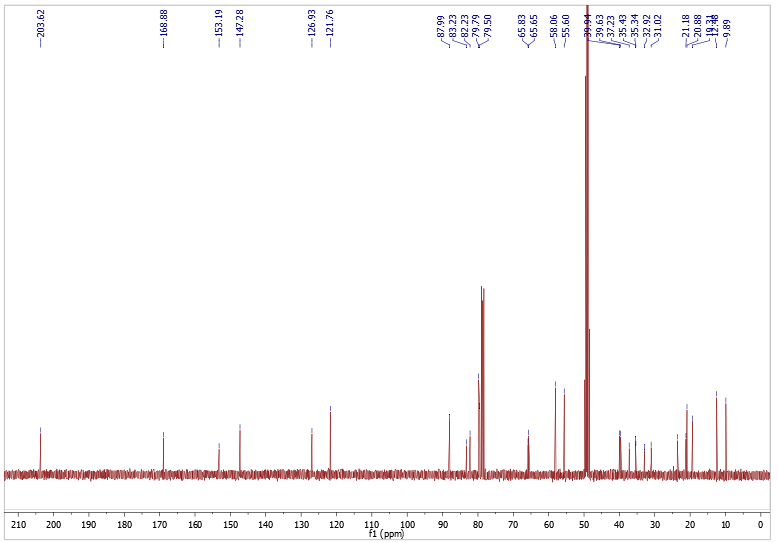


**Figure S9**. ^13^C NMR spectrum (100 MHz, CD_3_OD + 20% CDCl_3_) of compound **2**.

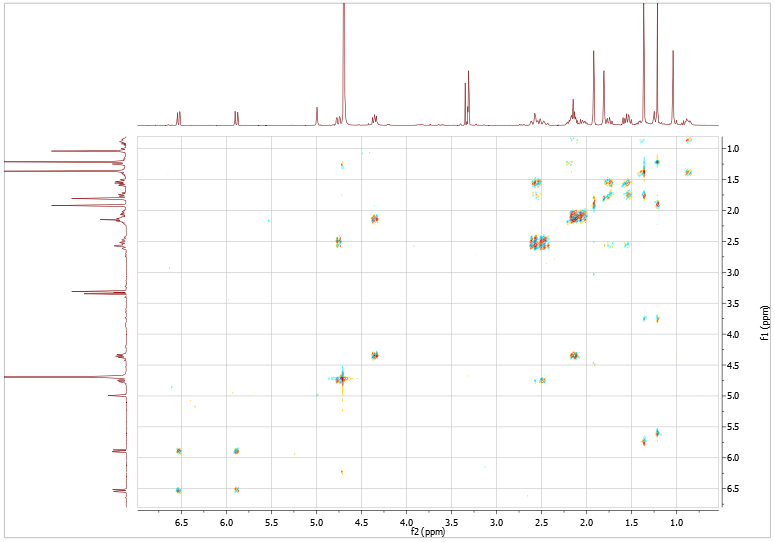


**Figure S10**. COSY NMR spectrum (400 MHz, CD_3_OD + 20% CDCl_3_) of compound **2**.

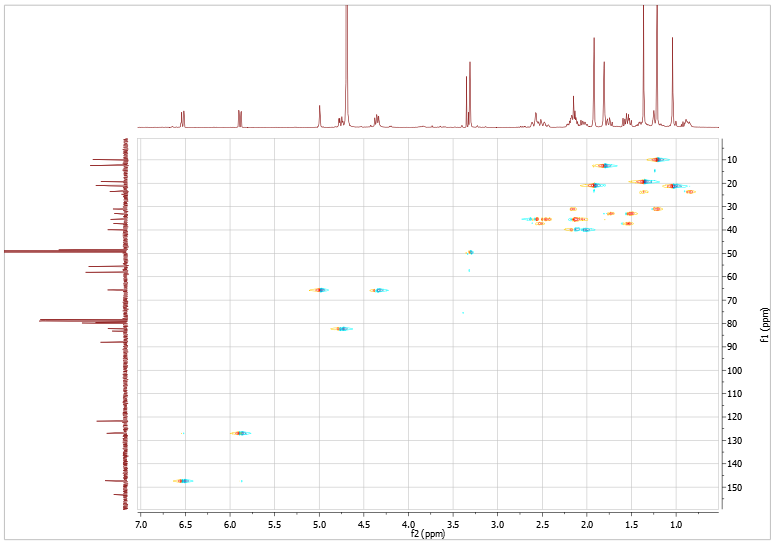


**Figure S11**. HSQC NMR spectrum (400 MHz, CD_3_OD + 20% CDCl_3_) of compound **2.**

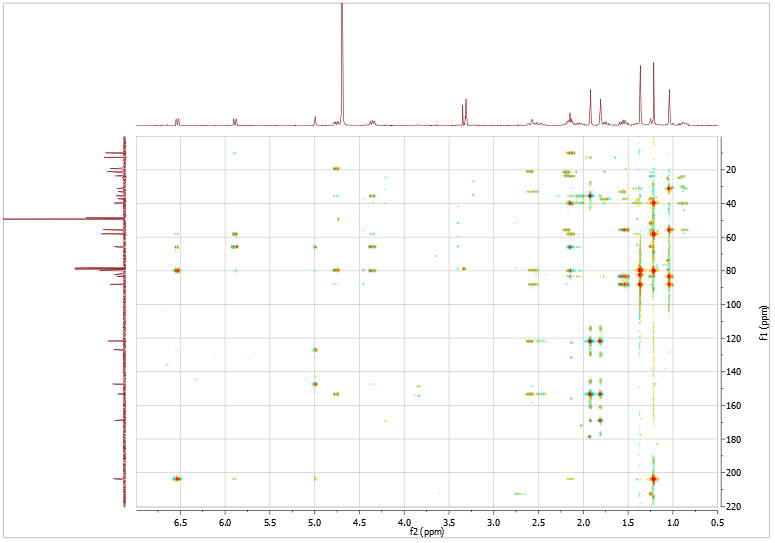


**Figure S12**. HMBC NMR spectrum (400 MHz, CD_3_OD + 20% CDCl_3_) of compound **2.**

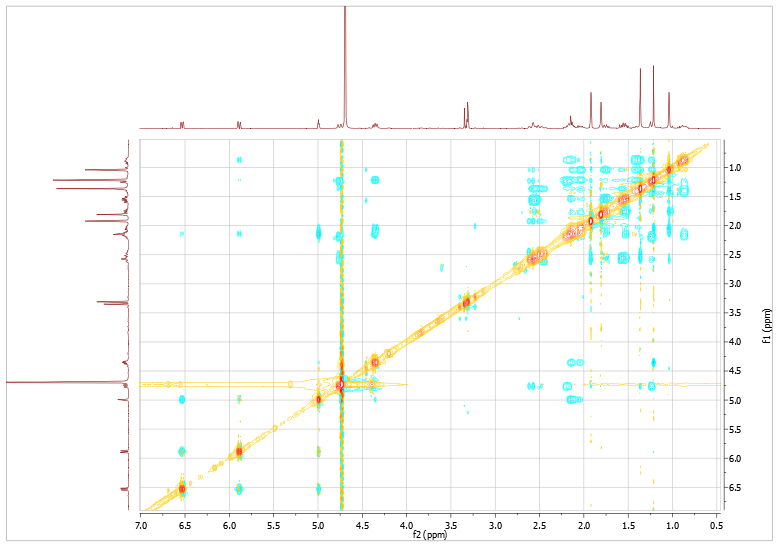


**Figure S13**. NOESY NMR spectrum (400 MHz, CD_3_OD + 20% CDCl_3_ to improved solubility) of compound **2**.


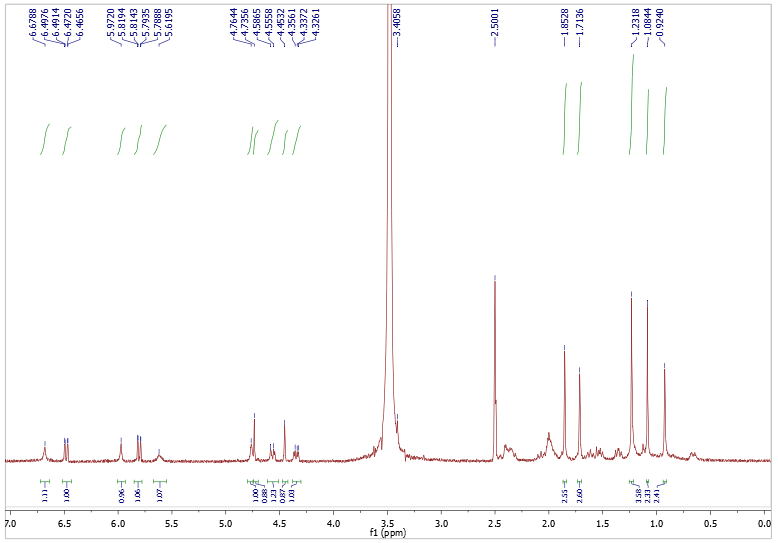


**Figure S14**. ^1^H NMR spectrum (400 MHz, DMSO) of compound **2**.

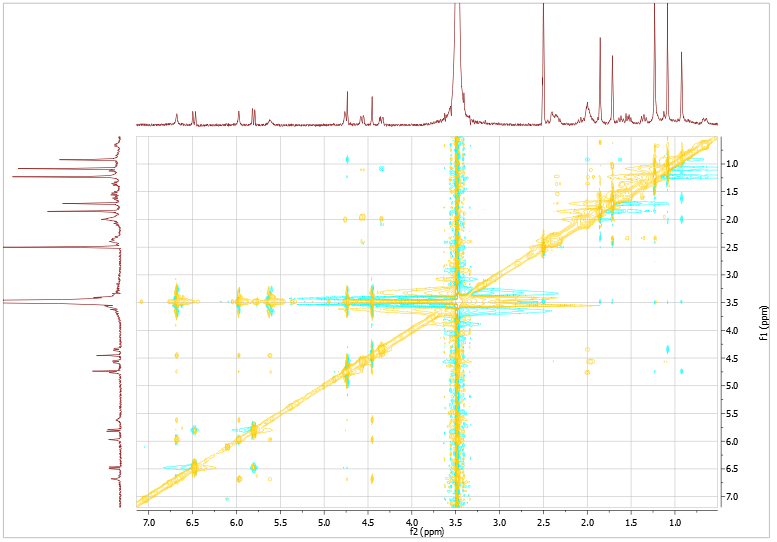


**Figure S15**. NOESY NMR spectrum (400 MHz, DMSO) of compound **2**.

**
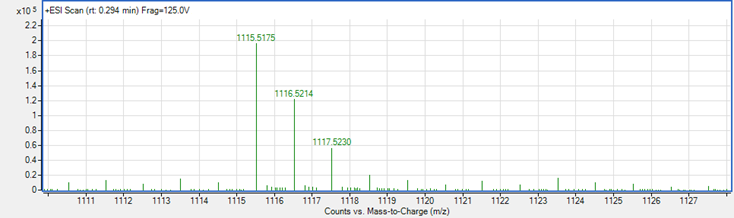
**

**Figure S16.** HR-ESIMS of compound **1**.

**
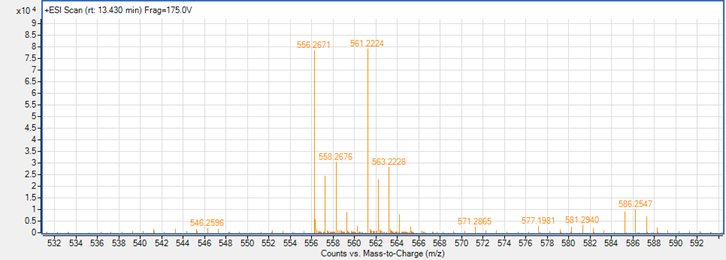
**

**Figure S17.** HR-ESIMS of compound **2**.
